# Clustering of Membrane Receptors: Insights from DNA Origami‐Based Approaches

**DOI:** 10.1002/smll.202503543

**Published:** 2025-06-03

**Authors:** Carmen M. Domínguez, Christof M. Niemeyer

**Affiliations:** ^1^ Institute for Biological Interfaces 1 (IBG‐1) Karlsruhe Institute of Technology (KIT) 76344 Eggenstein‐Leopoldshafen Germany

**Keywords:** cell signaling, clustering, DNA nanostructures, membrane receptors

## Abstract

Cell signaling enables cells to interpret and respond to their environment, relying on receptor interactions that regulate key biological functions. While receptor‐ligand affinity is crucial, receptor clustering plays a central role in modulating signaling efficiency, influenced by factors such as membrane diffusivity, lipid organization, and receptor‐receptor interactions. While advances in imaging have long contributed to a better understanding of this fundamental biological mechanism, recent breakthroughs in nanotechnology, particularly the use of DNA origami nanostructures (DONs), now enable the precise manipulation of receptor‐ligand interactions. This opens up unprecedented insights into the dynamics of signal transduction at the nanoscale and holds promising potential for innovative therapeutic applications. The examples presented in this article, based on various classes of receptors, illustrate how these groundbreaking developments can not only lead to a more detailed mechanistic understanding, but also pave the way for the development of new molecular therapies.

## Graphical abstract

Receptor clustering critically influences cell signaling, yet its precise regulation remains elusive. This review highlights how DNA origami structures serve as nanoscale tools to uncover receptor organization and provide new insights into signal transduction. Examples from various receptor classes highlight how this approach deepens mechanistic insight and informs new therapeutic strategies.

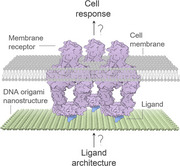

## 1 Brief Introduction to Receptor Clustering: Function and Importance for Cell Survival

Cell signaling is a fundamental process that enables cells to interpret and respond to their environment, facilitating communication, adaptation, and survival in dynamic biological contexts. This process typically involves biochemical—such as hormones, cytokines, or growth factors— or mechanical signals that target specific receptors on the cell surface, initiating a cascade of intracellular events that ultimately regulate key cellular functions and organismal homeostasis. While receptor‐ligand affinity is a central determinant of receptor interaction with ligands, receptor clustering within the cell membrane has long been recognized as a critical regulatory factor in the initial stages of signaling.^[^ ^1^ ^]^ Ligand engagement often induces receptors to transition from freely diffusing monomers to dimers and eventually to higher‐order oligomers. Notably, evidence suggests that even in the resting state,^[^ ^2^ ^]^ many receptors are not present as isolated monomers but are pre‐organized into nanoscale clusters. Given the central role of receptor clustering in maintaining cellular homeostasis, advancing methodologies that enable a mechanistic understanding of these complex processes is essential for effectively addressing their dysregulation in disease contexts.

When clustering is driven by multivalent ligand binding, receptor surface density also becomes a key factor in determining the efficiency of clustering, working reciprocally with the diffusivity. Specially, at low receptor densities—depending on the specific system—diffusivity becomes key for enabling effective multivalent interactions.^[^ ^3^ ^]^ Alternatively, receptor clustering may occur in lipid domains with reduced diffusion capability. Due to their spatial proximity, the entropic cost associated with ligand‐induced clustering is minimized, making the process energetically more favorable. This aligns with the historical notion that receptor clustering is associated to the existence of lipid rafts on the membrane.^[^ ^4^ ^]^ Beyond membrane lipid organization, it is increasingly evident that receptor clustering is significantly influenced by receptor‐receptor interactions through trans‐ or juxtamembrane domains, as well as by the structural organization of the actin cytoskeleton.^[^ ^5^ ^]^

Initial discoveries of receptor dimerization in the tyrosine kinase receptor superfamily^[^ ^6^ ^]^ laid the foundation for understanding clustering mechanisms. Today, receptor clustering is recognized as a critical regulatory feature in a variety of signaling receptors, including receptor tyrosine kinases (RTKs),^[^ ^7^ ^]^ immune receptors,^[^ ^4a^ ^]^ and cell adhesion molecules (CAMs) such as integrins and cadherins.^[^ ^8^ ^]^ In simple systems, basic advantages that receptor clustering confers for transmembrane signal transduction include increased ligand sensitivity driven by cooperative interactions,^[^ ^9^ ^]^ enhanced binding affinity due to higher local concentration and improved rebinding capabilities,^[^ ^10^ ^]^ as well as a broader dynamic range.^[^ ^11^ ^]^ In more complex systems, like the immunological synapse, receptor clustering enhances both sensitivity and specificity, allowing T cells to distinguish between self and non‐self antigens with an all‐or‐nothing response despite minimal affinity differences.^[^ ^12^ ^]^

Given the pivotal role of receptor clustering in maintaining cellular homeostasis, developing methodologies to achieve a mechanistic understanding of these complex cellular processes seems crucial for effectively addressing their dysfunction in disease contexts. The experimental investigation of receptor clustering is challenging due to both the nanoscale dimensions of the receptors and the dynamic nature of the interaction, which require high‐resolution imaging techniques capable of resolving structures in the range of tens to hundreds of nanometers while also capturing dynamic interactions in live‐cell environments. Advancements in receptor clustering research have been greatly driven by imaging microscopy techniques, particularly super‐resolution microscopy, combined with micropatterning methodologies on diverse display platforms, which include among others diblock copolymers,^[^ ^13^ ^]^ metal nanoparticles,^[^ ^14^ ^]^ supported lipid bilayers (SLBs),^[^ ^15^ ^]^ and multivalent immunogenic protein scaffolds.^[^ ^16^ ^]^ Such studies reveal that ligand properties, like spatial arrangement, valency, and mobility, play crucial roles in modulating cell signaling, activation, and effector functions. However, these systems lack precise control over the spatial parameters of ligand presentation, resulting in limited insight into the nanoscale requirements essential for receptor activation and the design of targeted therapeutic strategies. Fortunately, DNA nanotechnology has provided the field with DNA origami nanostructures (DONs), whose programmable and biocompatible nature enables the precise manipulation of receptor‐ligand interactions with unprecedented nanoscale control. Functional DONs are emerging as powerful tools for studying the biology of receptor clustering. While research in this field is still in its early stages, these systems have already demonstrated remarkable success by offering unparalleled control over ligand stoichiometry and spatial organization. They have been effectively applied to investigate various receptors, including immune receptors, RTKs, and integrins. In this review, we outline the methodologies for generating functional DONs and highlight recent studies that provide promising insights into receptor clustering processes. For a broader overview of diverse DNA nanotechnology approaches, including DNA origami as well as other DNA‐based platforms used to manipulate cell membrane receptors and modulate cell behavior, we refer the reader to the review articles by Fan et al. and Tseng et al.^[^ ^17^ ^]^

## 2 DNA Origami: Assembly Strategies for Ligand Presentation to Cells

**Figure 1:**
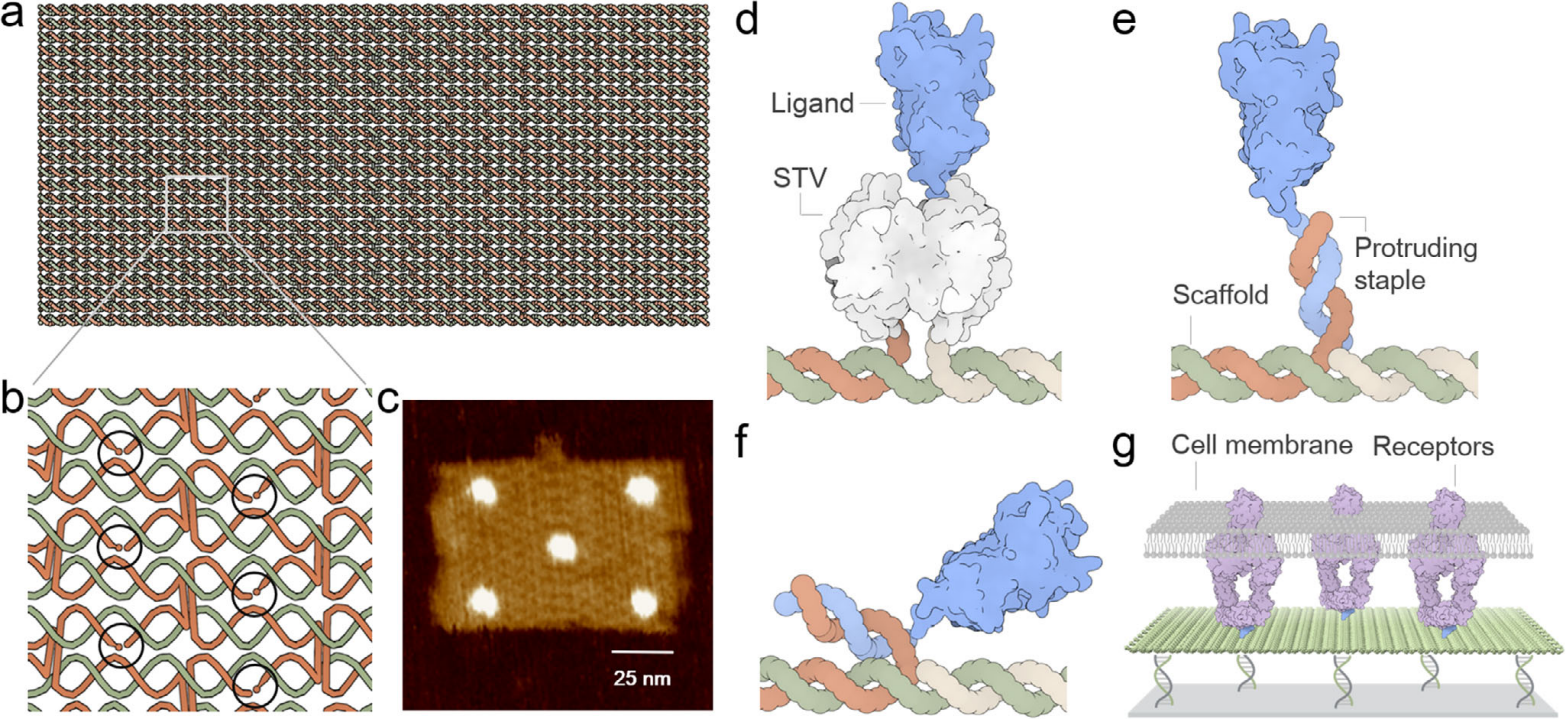
a) Schematic representation of a single‐layer square lattice DON.^[^ ^18^ ^]^ b) The scaffold strand is depicted in green and the staple strands are shown in orange. Black circles indicate positions that can be independently modified to bind ligands. c) Representative atomic force microscopy (AFM) image of a DON decorated with ligands at specific positions (own work). Note that this structure corresponds to the design shown in (a), although the Adenita representation displays a larger aspect ratio than it has in reality. d–f) Examples of common protein ligand decoration strategies for cell experiments. d) Two protruding staples incorporate biotin groups that bind STV, which in turn binds biotinylated ligands. e) Ligands are chemically coupled with DNA oligonucleotides that hybridize with strands protruding from the DON, with ligands extending outward (f) or with ligands oriented toward the DON. g) Schematic representation of a cell experiment where DONs with protruding staples on the lower plane are immobilized on a surface via DNA‐directed immobilization (DDI). Note that the DON can also be tethered to lipid bilayers using cholesterol‐modified staples or delivered in solution to cells, which eliminates the need for lower‐plane staples (not shown).

DNA origami technology relies on the bottom‐up fabrication of precisely defined quasi‐2D and 3D nanostructures by folding a long circular single‐stranded DNA molecule, the so‐called scaffold (typically 7.000 – 8.000 bases), with hundreds of short oligonucleotides known as staples (**Figure** **1**a,b).^[^ ^18^ ^]^ These staples, guided by computer‐aided design, contain multiple binding domains that hybridize with distant regions of the scaffold through crossover base pairing, folding the scaffold into the desired shape with high fidelity. While comprehensive reviews cover the design, assembly, and functionalization of DNA origami,^[^ ^19^ ^]^ the recent advances in isothermal annealing are particularly noteworthy in the context of the ligand‐modified origami structures discussed here. Typically, DNA origami formation is driven by self‐assembly under thermal annealing conditions, with assembly times ranging from hours to days, following a thermal denaturation step. Earlier isothermal approaches relied on denaturing agents or were limited to specific 2D structures.^[^ ^20^ ^]^ Recently, Rossi‐Gendron et al. demonstrated the isothermal assembly of a broad range of origami nanostructures in sodium‐based, magnesium‐free buffer.^[^ ^21^ ^]^ Mg^2+^ is traditionally used in DNA origami assembly and storage buffers due to its stabilizing effect. However, beyond its influence on yield and storage stability, it also affects the formation of undesired aggregates, possibly due to kinetically trapped states.^[^ ^22^ ^]^ This method prevents such complexes without thermal or chemical denaturation. While this approach may be slower and result in lower DNA origami yields, it offers the important advantage of enabling direct protein incorporation during folding—an essential feature for the applications discussed in this review.

The staples with unique sequences and positions act as addressable sites for attaching functional moieties, enabling the DON to serve as a pegboard for presenting biomolecular patterns to cells with full control of their arrangement at the nanoscale (Figure 1b,c). Temperature‐resistant ligands, such as small‐molecule hapten groups like 2,4‐dinitrophenyl (DNP), can be covalently attached to the staples and directly integrated into the DON during thermal annealing.^[^ ^23^ ^]^ On the contrary, temperature‐sensitive molecules like proteins are typically conjugated to the complex nanostructures after thermal annealing. In fact, the majority of studies using DONs for ligand presentation to cell receptors employ streptavidin (STV)/biotin bridges^[^ ^24^ ^]^ (Figure 1d). However, the attachment of STV to biotin groups on the DON surface may also be considered relevant for biological studies since direct coupling of STV to biotin groups lacking linkers can restrict access to the binding pockets of receptors, resulting in unexpectedly high reversible binding and lower receptor recruitment.^[^ ^24b^ ^]^ Alternatively, pre‐formed protein‐DNA conjugates can be hybridized with overhangs extending from the DON (Figure 1e–g).^[^ ^2^, ^24^, ^25^ ^]^ While these methods ensure the preservation of the protein function, they represent a trade‐off between the occupancy achieved and the precision of the localization on the DON. Longer overhangs can enhance both occupancy and ligand accessibility to receptor‐binding sites,^[^ ^25^, ^26^ ^]^ but may compromise the precision of ligand positioning and therefore, the receptor activation.^[^ ^24^, ^27^ ^]^ To enhance control over ligand positioning, Wang et al. designed the protein‐DNA conjugates, so that, upon hybridization, the ligand remained oriented toward the DON, near the site where the protruding strand originates (Figure 1f).^[^ ^25f^ ^]^ However, the high local concentration of negative charges in the DON could induce electrostatic repulsion in proximity to the negatively charged cell surface, potentially hindering receptor accessibility.^[^ ^26^ ^]^ It is important to emphasize that there is no universal rule for balancing spatial precision and flexibility in ligand presentation, as this depends heavily on the specific receptor under study. For instance, García‐Chamé et al. found that arrangements of RGD exposed via a 22‐mer linker favored integrin‐mediated cell migration. This is likely because integrins are bent in their inactive conformation, and the flexible linker enhances access to the integrin binding sites compared to a more rigid system based on STV‐biotin bridges.^[^ ^25c^ ^]^ Conversely, Comberlato et al. observed that including a linker between a CpG‐oligonucleotide ligand and the DON diminished TLR9 receptor activation, with progressively reduced activation correlating with increased linker length.^[^ ^27^ ^]^ A sophisticated strategy for decorating DONs with protein ligands is the use of self‐ligating protein tags, such as SNAP‐ or Halo‐Tag. While these tags have primarily been employed to anchor enzymes onto DONs for biocatalysis,^[^ ^28^ ^]^ SNAP tags have also been utilized to generate chimeric membrane receptors bearing an extracellular oligonucleotide that hybridizes with a complementary strand extending from the DON, facilitating receptor clustering.^[^ ^25d,g^ ^]^ Of note, in these setups, the length of the protruding staples can be precisely adjusted to tune the duration of the interaction (dwell time) as a measure of receptor‐ligand affinity.

Just as the choice of ligand functionalization strategy significantly impacts receptor clustering and subsequent cellular responses, the mode of DON presentation—whether immobilized on a surface or delivered in solution — must also be carefully considered, as it may influence receptor behavior and cellular responses. For such cell experiments, DONs with protruding staples on the bottom side can be immobilized on a surface via DDI (Figure 1g). For this purpose, single‐stranded capture oligonucleotides are covalently immobilized on various surfaces using chemical methods, often based on organosilane chemistry. These surfaces are typically composed of silica or glass, but can also include polymers. Such methods have been well established over decades in the context of DNA microarray development and allow for high surface densities. These functionalized surfaces can then be used for the specific immobilization of a wide range of components—including organic molecules, proteins, nano‐ and microparticles, DNA nanostructures and even whole cells—that have been pre‐functionalized with complementary oligonucleotides. Details on experimental protocols and the broad range of applications of the DDI method have been described in various review articles.^[^ ^29^ ^]^

Alternatively, the DON structure can be attached to lipid bilayers via hybridization to cholesterol‐modified staples or delivered in solution to cells, obviating the need for lower‐level staples. For readers interested in the various strategies used to anchor DNA nanostructures onto lipid surfaces, Tseng et al. provide a comprehensive discussion, covering factors that influence deposition yield, such as the immobilization method, nanostructure size, buffer ionic strength and linker length, among others.^[^ ^17b^ ^]^

In the case of the DDI‐based method, the use of microfluidic systems allows for a detailed characterization of binding and kinetics during surface decoration, which beneficially supports the execution and evaluation of cell experiments.^[^ ^24d^ ^]^ In research involving TCRs, DONs decorated with activating ligands are often presented on SLBs, allowing mobility of the DON modules and enabling them to be dragged by the receptor upon activation. Generally, TCR activation triggers actin network nucleation and retrograde flow, which transports TCR clusters toward the cell center.^[^ ^25d^ ^]^ Whether variations in DON diffusivity on SLBs influence immune receptor activation remains an open question.^[^ ^30^ ^]^ Similarly, and more broadly, direct comparisons of the responses of the same receptor to surface‐bound versus solution‐delivered DONs remain largely unexplored, highlighting a gap in understanding that is relevant across various biological contexts.

Another point of concern addresses the issue of DON degradation. When interfacing with cells, DONs are exposed to nuclease activity present in cell culture media, particularly in fetal bovine serum (FBS). Recent studies on minimal DNA nanocages have demonstrated degradation by extracellular nucleases in FBS, followed by internalization of cyanine dyes contained in the DNA structures.^[^ ^31^ ^]^ However, other works have assessed the stability of DONs in cell culture experiments using AFM, TEM, and agarose gel analysis, finding that degradation was not a significant issue.^[^ ^24^, ^25^ ^]^ This discrepancy may be due to the shorter incubation times and more complex DNA structures used in these experiments compared to the nanocage studies. Protective strategies, such as coating DONs with oligolysine‐PEG, have been explored but may result in reduced receptor‐binding affinity.^[^ ^2c^ ^]^ Alternative approaches include adding actin to the cell culture medium, which naturally inhibits DNase I, or using fully synthetic media lacking FBS.^[^ ^32^ ^]^ The latter options seem more straightforward and effective solutions for mitigating degradation issues in cell culture experiments involving DNA‐based materials; however, such approaches are not feasible for in vivo studies in animals, where additional stability and delivery challenges must be considered.

## 3 Applications of Biofunctional DONs to the Investigation of Receptor Clustering

With the aid of tools from DNA nanotechnology, it is now possible to investigate receptor clustering at the molecular level, addressing key questions such as the proximity and number of receptors required to form active clusters. Since immune receptors have recently been discussed in detail,^[^ ^17^, ^30^ ^]^ this section focuses on Fc receptors (FcRs), RTKs, integrins, and tumor necrosis factor receptors (TNFRs), all of which have been shown to form nanoclusters upon ligand binding or require multivalent ligand presentation for clustering and activation. It is worth noting that other receptors, whose activation is generally attributed to force‐induced conformational changes rather than clustering, can also be studied using DNA origami structures. In the case of the Notch receptor, e.g., a recent study proposes an alternative mechanism involving prolonged ligand engagement through multivalent presentation.^[^ ^33^ ^]^

### 3.1 Immune Receptors: FcγR and FcɛRI

Biofunctional ligand‐decorated DON systems have been widely employed to investigate clustering effects in immune receptor signaling. Receptors such as the T cell receptor (TCR) and Fc receptors (FcRs) elicit higher cellular activation when clustered with reduced intermolecular spacing.^[^ ^23^, ^25^ ^]^ In contrast, the B cell receptor (BCR) showed an inverse relationship, triggering greater activation with increasing clustering distances.^[^ ^34^ ^]^ Two recent reviews specifically address the use of DNA origami to investigate immune receptor activation, with a particular emphasis on the TCR.^[^ ^17^, ^30^ ^]^ Tseng et al.^[^ ^17b^ ^]^ further broaden this perspective by discussing DNA‐based nanostructures as immunogens or adjuvants in immunotherapy, as well as their potential for probing immune receptors and mediating immune cell‐cell interactions. In the following section, we will focus on recent advances related specifically to FcRs. These receptors bind to the Fc region of immunoglobulins, linking humoral responses to cellular activities. A subset of FcRs, predominantly expressed by leukocytes, mediates antibody effector functions. Most leukocyte FcRs are hetero‐oligomeric complexes and initiate a broad range of biological responses, which can be either activatory or inhibitory, upon cross‐linking.^[^ ^35^ ^]^

**Figure 2:**
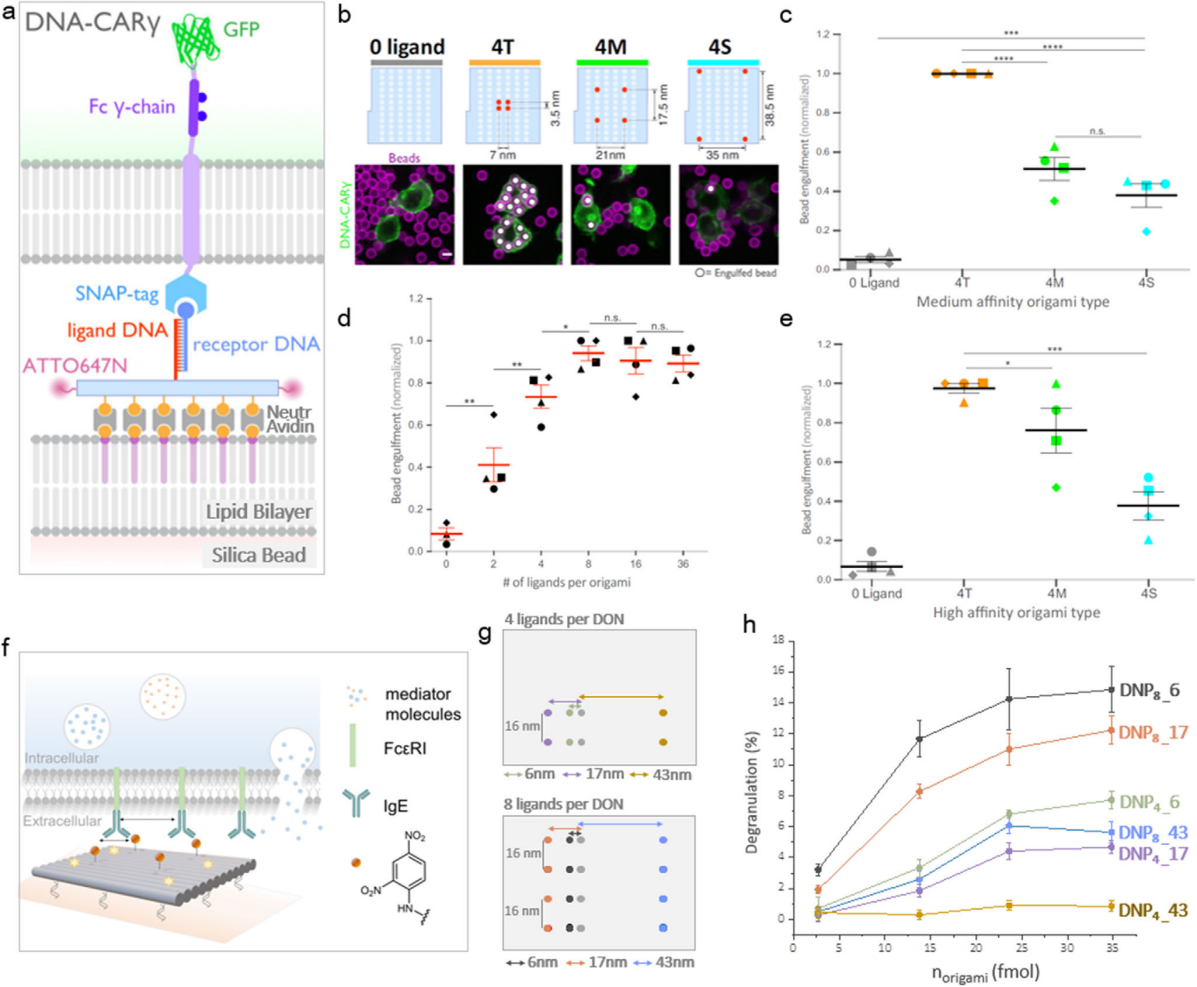
DON‐based study of FcγR (a–e).^[^ ^25g^ ^]^ a) Schematics of a cell line expressing a synthetic FcγR chimeric receptor, DNA‐CARγ receptor, in which the native extracellular ligand‐binding domain of FcγR was replaced with an extracellular SNAP‐tag that covalently binds a benzyl‐guanine labeled oligonucleotide (receptor DNA) used for assembly on DON. The receptor contains a transmembrane domain that is fused with the intracellular signaling domain of the FcγR chain, tagged with green‐fluorescent protein (GFP). After hybridization with the ligand DNA protruding from the DON, receptor clustering occurs. DONs are tethered to lipid bilayers that coat silica beads. b) DON designs showing no ligands (negative control) or 4 ligands distanced by 7 (4T), 21 (4M) or 35 nm (4S). The confocal microscopy images below show bead (bilayer in magenta) engulfment by macrophages (green). Internalized beads are indicated with white spheres. c) Bead phagocytosis experiments with the DON designs from (b). Ligand affinity is defined by the sequence of the ligand‐receptor DNA pair, with a hybridization dwell time of ≈24 s. d) Bead phagocytosis as a function of the number of ligands per origami, with intermolecular distances matching those in the 4T design. e) Bead phagocytosis with the DON designs from (b), but with increased ligand affinity, resulting in a hybridization dwell time exceeding 7 h. Adapted with permission.[22h]. DON‐based study of FcɛRI (f–h).^[^ ^23a^ ^]^ f) Schematic of a degranulation experiment using mast cells expressing FcɛRI receptors. DONs functionalized with DNP ligands were used. Cells were sensitized with anti‐DNP IgE prior to exposure to DON functionalized surfaces. The degranulation response was quantified biochemically using a β‐hexosaminidase assay. g) DON designs featuring either 4 or 8 ligands per DON. Ligands are arranged in vertical pairs to enable bivalent IgE binding, with an optimal intermolecular distance of 16 nm for the bivalent interactions. Horizontal distances for receptor clustering are set to 6, 17, and 43 nm. h) Degranulation response of anti‐DNP IgE‐sensitized RBL‐2H3 cells following incubation on immobilized DNP‐DON as shown in (g). Adapted with permission.^[^ ^23a^ ^]^

IgG is recognized and bound by FcγR on macrophages, dendritic cells (DC), and other immune cells, facilitating various immune effector functions, including antibody‐dependent cellular phagocytosis. Using silica nanoparticles coated with SLBs containing the decorated DONs (**Figure** **2**a), Kern et al. demonstrated that tightly spaced ligands (≤7 nm) significantly enhanced both the likelihood and kinetics of phagocytosis compared to the same number of more dispersed ligands (>20 nm) (Figure 2b,c).^[^ ^25g^ ^]^ They also observed that increasing the number of ligands led to a boost in phagocytosis, reaching a plateau at 8 ligands per cluster (Figure 2d). Notably, their data ruled out an avidity effect, as ligand spacing influenced phagocytosis independently of receptor‐ligand affinity (Figure 2e). Within the FcR family, the immunoreceptor tyrosine‐based activation motif (ITAM) is critical for initiating downstream signaling pathways. Upon FcγR crosslinking, Src protein tyrosine kinases (PTKs) phosphorylate the ITAM, that becomes a docking site for Syk, a PTK that transmits a signal leading to actin polymerization and particle uptake.^[^ ^36^ ^]^ Indeed, tight receptor clustering was shown to also enhance receptor phosphorylation and Syk recruitment.^[^ ^25g^ ^]^ The authors proposed that this nanoscale clustering enables macrophages to distinguish highly opsonized targets from low‐density signals, such as soluble antibodies. Regarding soluble formats, Douglas et al.^[^ ^37^ ^]^ recently demonstrated phagocytosis of antibody‐labeled 3D DONs by macrophages and DC. Uptake by DCs was valency‐dependent, likely due to their higher expression of FcγRI, which strongly interacts with the specific IgG used. The study compared different 3D DONs, barrels (30 nm × 60 nm) and rods (7 nm × 400 nm), and found that macrophage uptake of barrels showed minimal valency dependence, whereas uptake of rods increased with higher antigen valency, suggesting that the 3D shape of the DON influences FcγR‐mediated uptake sensitivity.

Most effector leukocytes co‐express multiple FcγRs, including activating receptors bearing the above mentioned ITAMs (e.g., FcγRI, FcγRIIa, FcγRIIc, FcγRIIIa) and the single inhibitory receptor FcγRIIb, which contains an immunoreceptor tyrosine‐based inhibition motif (ITIM).^[^ ^38^ ^]^ The interplay between activating and inhibitory signals establishes a critical threshold for IgG‐mediated immune responses.^[^ ^38^ ^]^ Furthermore, activating FcγRs can also generate inhibitory signals when engaged at low level^[^ ^39^ ^]^ or depending on the specific properties of the ligand.^[^ ^40^ ^]^ It is plausible that the multivalent nature of the interaction, including factors such as ligand valency and receptor cluster density, may influence whether a given FcγR elicits an activating or inhibitory response.^[^ ^40^ ^]^ However, how variations in receptor engagement modes lead to opposing functional outcomes, and how cells integrate conflicting signals from co‐expressed receptors to produce a coordinated response, remain open questions. DNA origami structures are particularly well‐suited to address these challenges, as they allow for precise control over both ligand composition and spatial arrangement. This enables the design of highly specific cellular triggers, whose downstream effects can be systematically analyzed, e.g., through transcriptomic profiling.

FcɛRI binds IgE with high affinity and is expressed on various immune cells, including mast cells. Clustering of these receptors likely provides an advantageous way for precisely detecting repetitive epitope patterns found on parasites or tumor cells overexpressing tumor antigens allergens, but also allergens, which can trigger undesired excessive immune system activation.^[^ ^41^ ^]^ Due to the high affinity binding, FcɛRI stably associates with monomeric IgE on the mast cell surface, enabling antigen recognition, receptor crosslinking, and the subsequent release of inflammatory mediators, including preformed granules, lipid mediators, and cytokines. Upon receptor engagement, the ITAM motif is phosphorylated by Lyn, a Src family PTK. This phosphorylation leads to the activation and recruitment of Syk PTKs, initiating downstream signaling pathways.^[^ ^35^ ^]^ Using DNP‐decorated DONs, Schneider et al. investigated FcɛRI clustering in RBL‐2H3 cells (Figure 2f).^[^ ^23a^ ^]^ It was found that optimal inter‐ligand distances for antibody binding (16 nm) resulted in higher activation when inter‐receptor distances were reduced (6 nm > 17 nm > 43 nm), as evidenced by increased β‐hexosaminidase release from RBL cell granules (Figure 2g,h). This trend aligns well with findings for FcγR reported by Kern et al.^[^ ^25g^ ^]^ Again, a clear valency effect was observed, translating in higher degranulation when using clusters of 8 ligands compared to the same intermolecular spacing of ligands yet a lower valency of 4. As the valency was reduced from 8 to 4, the need for closely spaced ligands became more critical, as clusters of 4 ligands separated by 43  nm were unable to effectively induce degranulation, regardless of the DON concentration. Interestingly, the nanoscale spacing effect was observed to exceed the valency effect, as e.g. 4 IgE ligands spaced by 6 nm elicited more potent degranulation than 8 ligands spaced by 43 nm (Figure 2h).

Altogether, these findings suggest that cells use FcRs clustering to discriminate between different extracellular signals, enhancing specificity and sensitivity in immune responses. This highlights the importance of nanoscale spatial organization in fine‐tuning immune receptor activation, as tightly spaced ligands (≤7 nm) significantly enhance effector functions mediated by FcγR and FcɛRI compared to more dispersed ligands, with valency also playing a crucial role in receptor activation.

**Figure 3:**
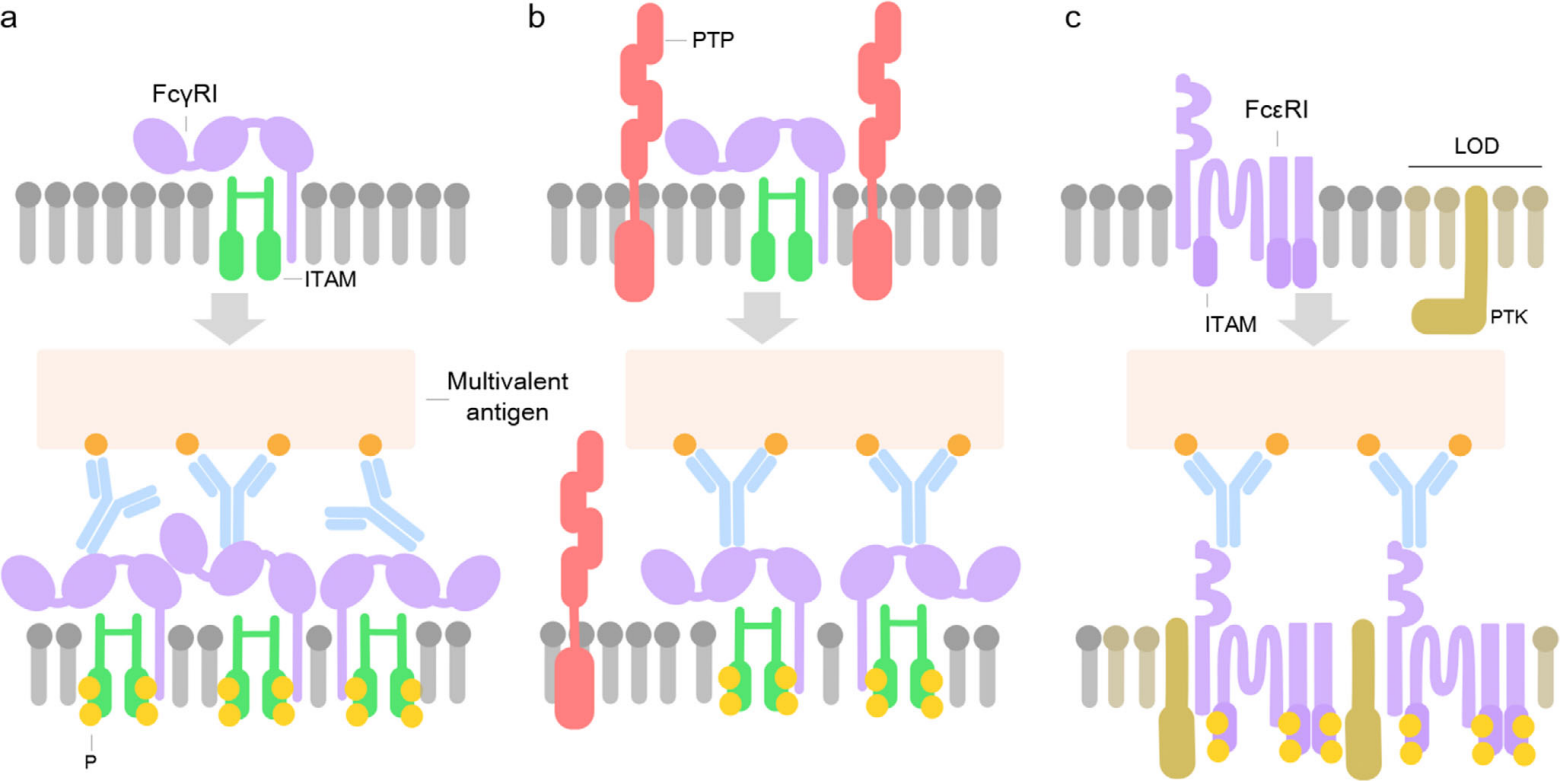
Schematic depiction of the hypothesized mechanisms that explain why dense‐ligand clustering promotes receptor phosphorylation: a) clustering allows achieving a critical intracellular concentration of ITAM domains; b) bulky phosphatases (PTP) are excluded from the interacting area; c) receptor clusters stabilize liquid ordered domains (LOD) where protein kinases are located (PTK).

The mechanism by which dense‐ligand clustering promotes receptor phosphorylation remains incompletely understood, though several hypotheses have been proposed. Given the similarities between FcγR and FcɛRI systems, it seems reasonable to discuss potential mechanisms together. Among the mechanisms proposed by Kern et al.^[^ ^25f^ ^]^ for the enhanced FcγR receptor activation upon tight clustering that may also apply to FcɛRI are: (i) this clustering may help achieve a critical intracellular concentration of ITAM signaling domains (**Figure** **3**a); and (ii) may enhance the exclusion of protein tyrosine phosphatases (PTPs) that dephosphorylate the FcRs or the Src PTKs and therefore allow sustained phosphorylation of the ITAM motifs (Figure 3b). Evidence suggests that the large phosphatase CD45, which contributes to dephosphorylation of FcγR and whose size prevents it from being included in the tight cell‐cell contacts mediated by IgG‐FcγR,^[^ ^42^ ^]^ is segregated from FcγR clusters.^[^ ^36^ ^]^ With respect to FcɛRI, findings suggest that upon antigen‐mediated crosslinking, FcɛRI clusters associate and stabilize liquid‐ordered like domains, preferentially excluding PTPs and promoting Lyn‐mediated phosphorylation (Figure 3c).^[^ ^43^ ^]^

Despite significant progress, key aspects of FcεRI signaling remain poorly understood. For instance, different antigen properties, such as low versus high affinity, have been shown to trigger qualitatively distinct mast cell responses. This suggests that FcεRI dynamics may translate antigen characteristics into specific signaling outcomes.^[^ ^44^ ^]^ Internalization of FcεRI further contributes to signal termination and cellular desensitization.^[^ ^44^ ^]^ However, it remains unclear how specific features of antigens, including valency, epitope spacing, and affinity, influence FcεRI cluster size, mobility, and internalization kinetics, or how these dynamic receptor behaviors are mechanistically linked to downstream outcomes such as degranulation, cytokine production, or desensitization. Addressing these open questions requires molecular tools that allow precise control over ligand composition and spatial parameters. DONs provide such capabilities, making them ideally suited to systematically probe how cells interpret complex antigen patterns to generate defined functional responses.

### 3.2 RTKs: Eph Receptors, Epidermal Growth Factor Receptor (EGFR), and Insulin Receptor (IR)

**Figure 4:**
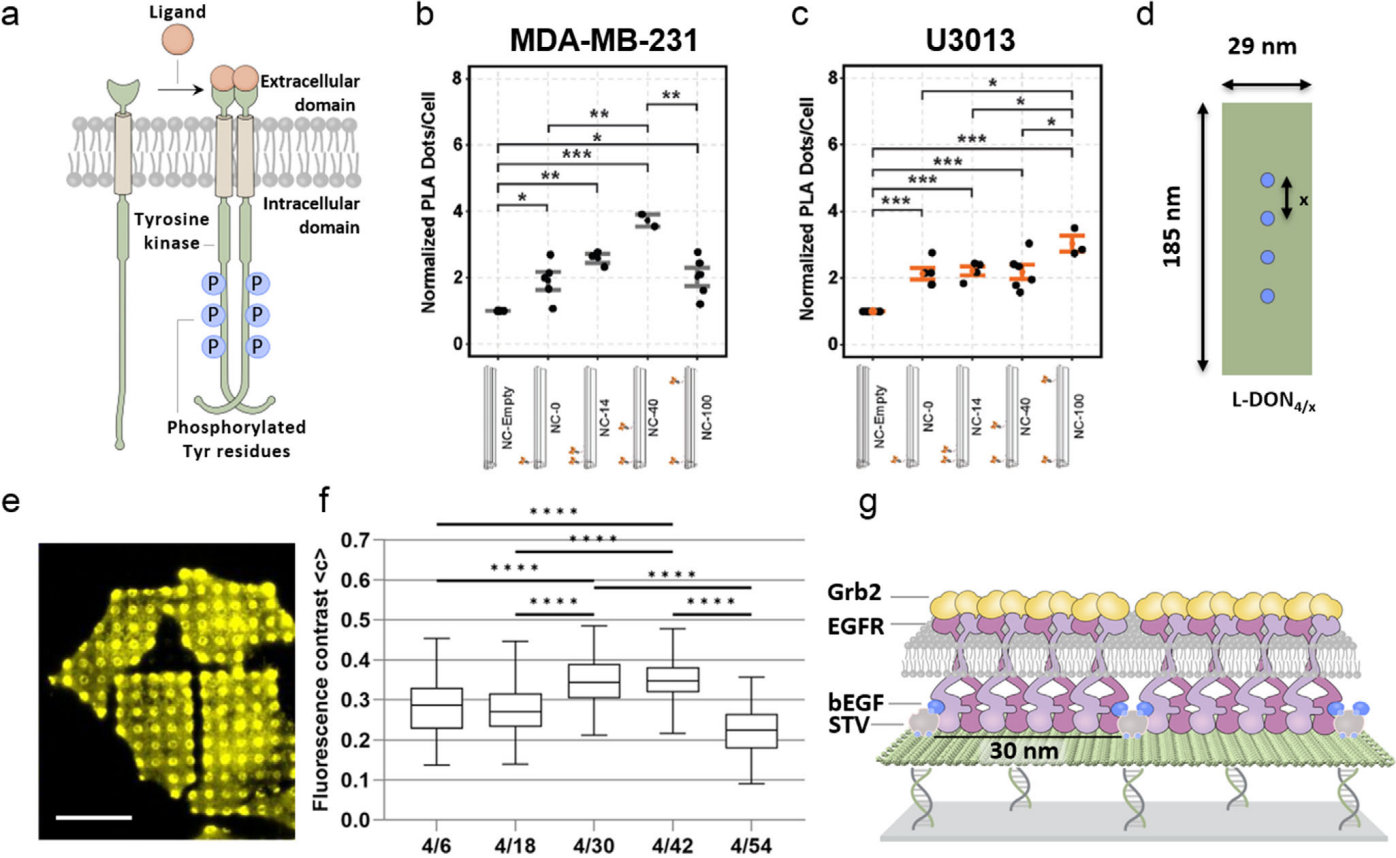
a) General structure of an RTK going through dimerization and transphosphorylation upon ligand binding. b,c) **DON‐based study of Eph receptors**.^[^ ^25b^ ^]^ Quantification of phosphorylated EphA2 receptor in b) MDA‐MB‐231 and c) U3013 cells, as determined by an in situ proximity ligation assay.^[^ ^49^ ^]^ Cells were treated for 15 min with soluble DON containing two ephrin‐A5 dimers distanced by 14, 40, or 100 nm (NC14, NC40 or NC100) and controls of empty DON (NC‐empty) or DON with only one single dimer (NC0). Reproduced with permission.^[^ ^25b^ ^]^ d–g) **DON‐based study of EGFR**.^[^ ^24c^ ^]^ d) Schematic depiction of DON ruler immobilized via DDI in micropatterns on glass surfaces. The inter‐ligand distances (x) were set to 6, 18, 30, 42, and 54 nm. e) TIRF microscopy image of Hela cells stably expressing Grb2‐YFP grown on surfaces functionalized with EGF‐decorated DON. Scale bar: 30 µm. f) Quantification of clustered Grb2 via Grb2‐YFP fluorescence contrast obtained with DON rulers decorated with four EGF ligands at variable distances. Adapted with permission.^[^ ^24c^ ^]^ g) A schematic illustration presenting the hypothesis that activation propagates laterally in accordance with the inter‐ligand spacing on the DON.

RTKs regulate crucial cellular processes such as survival, proliferation, and migration; and mutations or irregularities in RTKs are linked to various diseases. Despite their diversity, all RTKs share a similar structure: a single transmembrane region connects conserved intracellular kinase domains to variable extracellular ligand‐binding domains (**Figure** **4**a). Typically, activation of RTKs by ligand binding leads to receptor dimerization. This dimerization aligns intracellular domains, activating their kinase activity through trans‐phosphorylation, which then recruits downstream signaling proteins.^[^ ^45^ ^]^ In this section, we will focus on specific RTKs, including Eph receptors, the EGFR, and the IR.

Eph receptors are the largest class of RTKs, comprising 14 members classified as EphA and EphB in humans. Eph receptor signaling is critical for numerous developmental processes, guiding cells to specific positions through interactions with their membrane‐bound ephrin ligands, and is frequently dysregulated in cancer.^[^ ^46^ ^]^ They bind to ephrin ligands on the membrane of neighboring cells, triggering Eph/ephrin intracellular signaling pathways that mediate cell‐cell communication. Of note, Eph receptor activation may inhibit cell proliferation and migration and show tumor‐suppressive effects.^[^ ^47^ ^]^ In the foundational study by Shaw et al. in 2014,^[^ ^25a^ ^]^ soluble DONs were used to modulate the nanoscale spatial organization of ephrin‐A5 (≈26 kDa for the extracellular domain^[^ ^48^ ^]^) dimers and thus drive EphA2 receptor clustering in human breast cancer MDA‐MB‐231 cells. Specifically, an inter‐ligand distance of ≈40 nm (NC‐40) induced more efficient EphA2 receptor phosphorylation than a distance of ≈100 nm (NC‐100), resulting in reduced cell invasiveness compared to the negative control. In a follow‐up work,^[^ ^25b^ ^]^ the receptor phosphorylation with nanoscale distances was hypothesized to also depend on receptor expression levels. Unlike the results observed in MDA‐MB‐231 cells, human glioblastoma U3013 cells, with lower EphA2 expression, showed higher phosphorylation levels when stimulated with the NC‐100 DON as compared to the NC‐40 (Figure 4b,c). These DONs selectively activated the EphA2 signaling transcriptome in U3013 cells. Stimulation with NC‐40 influenced pathways associated with canonical EphA2 activation, including phosphatidylinositol, MAPK and NOTCH1 signaling. In contrast, NC‐100 primarily affected pathways downstream of those affected by NC‐40 stimulation, such as those related to cell cycle regulation. Notably, MDA‐MB‐231 cells exhibited minimal EphA2‐mediated transcriptome responses despite showing differential receptor activation depending on spatial arrangement. These studies demonstrate that the nanoscale spatial organization of ephrin ligands significantly influences EphA2 receptor clustering, phosphorylation, downstream signaling and invasive properties of cancer cells, with effects varying by cell type and likely receptor expression levels.

As discussed above, activation of Eph receptors can have tumor‐suppressive effects. However, Eph signaling is highly complex and often context‐dependent, leading to apparently paradoxical outcomes. The same interaction between an Eph receptor and its ephrin ligand can result in opposite cellular responses, such as repulsion versus attraction or inhibition versus promotion of cell migration, depending on the cellular environment. Consequently, Eph receptors have been linked to both tumor‐suppressive and tumor‐promoting processes.^[^ ^46^, ^50^ ^]^ Despite extensive research, the molecular mechanisms underlying these divergent signaling outcomes remain poorly understood. In particular, the influence of Eph receptor clustering on signal strength, duration, and specificity is still unclear. DNA tools offer precise spatial control over ligand presentation, enabling the design of highly specific cellular triggers whose downstream effects can be systematically analyzed, e.g., through omics profiling.

The EGFR is a member of the ErbB family of RTKs involved in oncogene signaling. Shortly after the foundational work on Eph receptors by Högberg, Teixeira and coworkers,^[^ ^25a^ ^]^ the Niemeyer group published a proof‐of‐concept study using biofunctional DONs on surfaces to investigate EGFR clustering.^[^ ^24a^ ^]^ In this study, micropatterned glass surfaces were used to present DONs decorated with epidermal growth factor (EGF, ≈6 kDa) in varying stoichiometries, either evenly distributed or densely clustered, to MCF‐7 cells. Immunostaining of phosphorylated EGFR indicated that EGFR clustering over the decorated spots led to receptor activation, and quantification of colocalized DON and activated EGFR spots suggested that nanoscale ligand architecture influences the cellular response. The number of activated spots per cell, and therefore the spreading of the cell, increased with ligand number and with sparsely distributed ligands (≈30–80 nm) compared to densely clustered ligands (≈6–20 nm). These findings were later corroborated by Mayer et al.,^[^ ^24c^ ^]^ where specific distances were systematically investigated using DON‐based molecular rulers (Figure 4d). Fluorescence microscopy‐based quantification of clustered Grb2 (Figure 4e), which directly binds to phosphorylated tyrosine residues on EGFR via its SH2 domain, revealed that maximum activation occurred at inter‐ligand distances of 30–42 nm. This was determined across tested distances ranging from 6 to 54 nm (Figure 4f). Based on previous findings these results contributed to the working hypothesis that EGFR activation can laterally propagate to neighboring unliganded EGFR molecules in the membrane.^[^ ^50^ ^]^ Although EGFR clustering does not inherently require multivalent ligand interactions, its activation by nanoscale EGF patterns can propagate laterally, promoting the formation of more stable oligomers. The better the alignment of ligand patterns with the receptor's naturally preferred oligomeric arrangements, the greater the stability of membrane clusters and the higher the recruitment of Grb2 (Figure 4g). Under the study's conditions, EGFR oligomerization predominantly resulted in the formation of octamers and decamers spanning 30–40 nm. This is consistent with findings by Needham et al.,^[^ ^51^ ^]^ who used fluorophore localization imaging with photobleaching to examine transient EGFR oligomers, suggesting that interactions between EGFR dimers (≈11 nm in diameter) could generate oligomers up to 50–60 nm in diameter at physiological EGF concentrations.

Since these studies demonstrated that EGFR activation and downstream signaling are highly sensitive to the nanoarchitecture of ligands—showing optimal activation at ligand spacings of 30–42 nm, likely due to lateral propagation and the formation of stable oligomers—we aim to further explore this working hypothesis in light of the findings on Eph clustering by Verheyen et al.,^[^ ^25b^ ^]^ (see Figure 4b,c). Lateral propagation of the phosphorylation signal by activated Eph receptors, mediated through direct Eph–Eph interactions rather than ephrin contact, has been reported previously.^[^ ^52^ ^]^ Given the approximate diameter of an Eph monomer (≈5–6 nm),^[^ ^53^ ^]^ it seems plausible that under the Verheyen study's conditions, and depending on the specific cell line, lateral propagation of Eph activation could result in oligomers spanning ≈40 or even up to ≈100 nm. This would align with findings by Ojosnegros et al., who noted that ephrin stimulation leads to maximal activation when low‐order oligomers formed by lateral recruitment predominate.^[^ ^54^ ^]^

To place the above hypothesis on molecular mechanisms into a general context, it is important to note that RTKs are often activated by a diverse range of ligands, each preferentially triggering specific signaling pathways and producing distinct functional outcomes—a phenomenon known as biased agonism.^[^ ^7^ ^]^ While the mechanisms underlying biased agonism are not fully understood, it was proposed for EGFR signaling over a decade ago that different ligands stabilize distinct EGFR dimer conformations, resulting in varying dimer lifetimes.^[^ ^55^ ^]^ Furthermore, we hypothesize that different ligands could possibly modulate oligomeric EGFR states by stabilizing distinct structural conformations, thereby shaping diverse signaling outcomes. In the case of Eph receptors, studies have indeed shown that different ligands stabilize structurally distinct oligomers formed through alternative interfaces. These unique oligomeric assemblies can exhibit distinct signaling properties, thereby contributing to the functional diversity of Eph receptor activity.^[^ ^56^ ^]^ Hence, RTK clustering may be a crucial mechanism for enhancing signaling specificity, in addition to regulating sensitivity and responsiveness to widely varying ligand concentrations and gradients, as previously proposed.^[^ ^54^, ^57^ ^]^ However, this hypothesis has yet to be confirmed. With DON‐based techniques now enabling precise variations in defined ligand architectures, including combinations of different ligands, there is an unprecedented opportunity to thoroughly investigate these mechanisms.

Understanding the fundamental mechanisms of receptor clustering could have direct implications for medical treatment. E.g., recent work by García‐Chamé et al. demonstrated that DON‐mediated EGFR clustering and activation is facilitated by the inhibitory antibody Panitumumab and is influenced by inter‐ligand spacing.^[^ ^24d^ ^]^ Notably, in EGFR, activating ligands do not directly contribute to the dimerization interface; instead, oligomerization is entirely receptor‐mediated.^[^ ^45^ ^]^ Previous studies have shown that inhibitory antibodies like Cetuximab and Matuzumab can induce EGFR phosphorylation through receptor dimerization and kinase activation although they do not activate the Ras/MAPK pathway.^[^ ^58^ ^]^ Since EGFR signaling primarily operates through two major pathways—one involving phosphoinositide 3‐kinase (PI3K) and Akt, and the other mediated by Ras, Raf, and Erk (MAPK)—future research should explore how DON‐mediated activation influences these downstream pathways to better assess their potential for therapeutic approaches.

Furthermore, EGFR signaling does not occur in isolation but is embedded within a complex network of interacting pathways. Crosstalk between EGFR and other signaling systems is increasingly recognized as a critical factor influencing tumor biology and therapeutic responses.^[^ ^59^ ^]^ However, the precise molecular mechanisms underlying many of these interactions, including the role of receptor clustering, remain incompletely understood. Notably, upon ligand binding and activation, EGFR undergoes internalization and is trafficked through early endosomes, where it can continue to signal.^[^ ^60^ ^]^ Whether the signaling complexes and downstream pathways activated in early endosomes differ qualitatively from those initiated at the plasma membrane is still not fully understood. How compartmentalization and receptor clustering within these intracellular compartments influence signal duration and specificity remains an open question. Adding further complexity, EGFR can also translocate from endosomes or the plasma membrane to other intracellular organelles, including the nucleus.^[^ ^61^ ^]^ Whether the spatial organization of EGFR clusters contributes to these processes remains to be elucidated. DONs enable precise ligand presentation, resulting in tailored nanoscale architectures that serve as powerful tools for generating specific cellular triggers. These engineered inputs allow systematic investigation of downstream signaling responses using a range of omics approaches, including transcriptomics, proteomics, phosphoproteomics, and metabolomics, providing deep insights into how cells interpret spatially organized molecular cues.

The IR is another RTK known to form nanoclusters on the membrane of adipocytes, β‐cells or hepatocytes. Binding of insulin (polypeptide hormone of 5,8 kDa) to the IR initiates a signaling cascade that culminates in glucose uptake into cells, lipogenesis and other anabolic processes.^[^ ^62^ ^]^ Spratt et al. decorated DON‐based nanorods (NR) with insulin conjugates and the reagents were tested on adipocytes to investigate the effects of multivalent insulin presentation.^[^ ^2c^ ^]^ Among the tested valencies (1, 2, 4, 7, and 15 insulin molecules per NR), the NR with 7 insulin molecules (NR‐7), featuring an approximate ligand spacing of 17 nm, elicited the strongest activation of the IR pathway. This was evidenced by elevated phosphorylation levels of IR and the downstream protein AKT, as quantified by Western blotting. The authors suggested that both insulin valency and ligand spacing regulate IR activation, as lowering either parameter resulted in reduced receptor activation. Analysis of transcriptional responses in adipocytes showed that NR‐7 induced >2000 differentially expressed genes compared to monovalent insulin display. Notably, the transcriptional response to NR‐7 peaked at 10 nm insulin, a concentration where pure insulin or monovalent‐decorated DON elicited minimal effects. This finding underscores the enhanced potency of multivalent insulin presentation in activating IR signaling and downstream transcriptional responses. The NR‐7 nanostructure was also evaluated in a zebrafish model lacking β‐cells, demonstrating its ability to lower free glucose levels. Given that IR dimers are ≈12 nm wide, the authors suggest that the ≈17 nm spacing between insulin ligands on NR‐7 aligns optimally with the spacing of IR nanoclusters on the cell membrane, maximizing avidity effects. In contrast, decreasing the inter‐ligand spacing or the number of ligands per NR may result in unbound insulin or unengaged receptors, respectively, thereby reducing receptor activation efficiency.

### 3.3 CAMs: Integrins

Integrins are heterodimeric receptors that link the actin cytoskeleton to the extracellular matrix (ECM), playing a critical role in cell adhesion, migration, invasion, and cancer progression.^[^ ^63^ ^]^ Their activation involves conformational changes from an inactive to an active state, enabling high‐affinity interactions with ECM ligands. Clustering of activated integrins, facilitated by the recruitment of anchoring and adapter proteins, leads to the formation of focal adhesions (FAs), 3D structures essential for integrin function. Receptor clustering enhances binding avidity, enabling ECM rigidity sensing and environmental assessment during early cell spreading. This process enables the rapid formation of nascent adhesions (precursors of FAs). Actin polymerization then mobilizes these clusters, supporting matrix rigidity sensing and dynamic adjustments in adhesion strength. By clustering receptors with weaker affinities, cells achieve strong but reversible adhesions, facilitating rapid turnover essential for efficient adhesion dynamics.^[^ ^8^ ^]^

**Figure 5:**
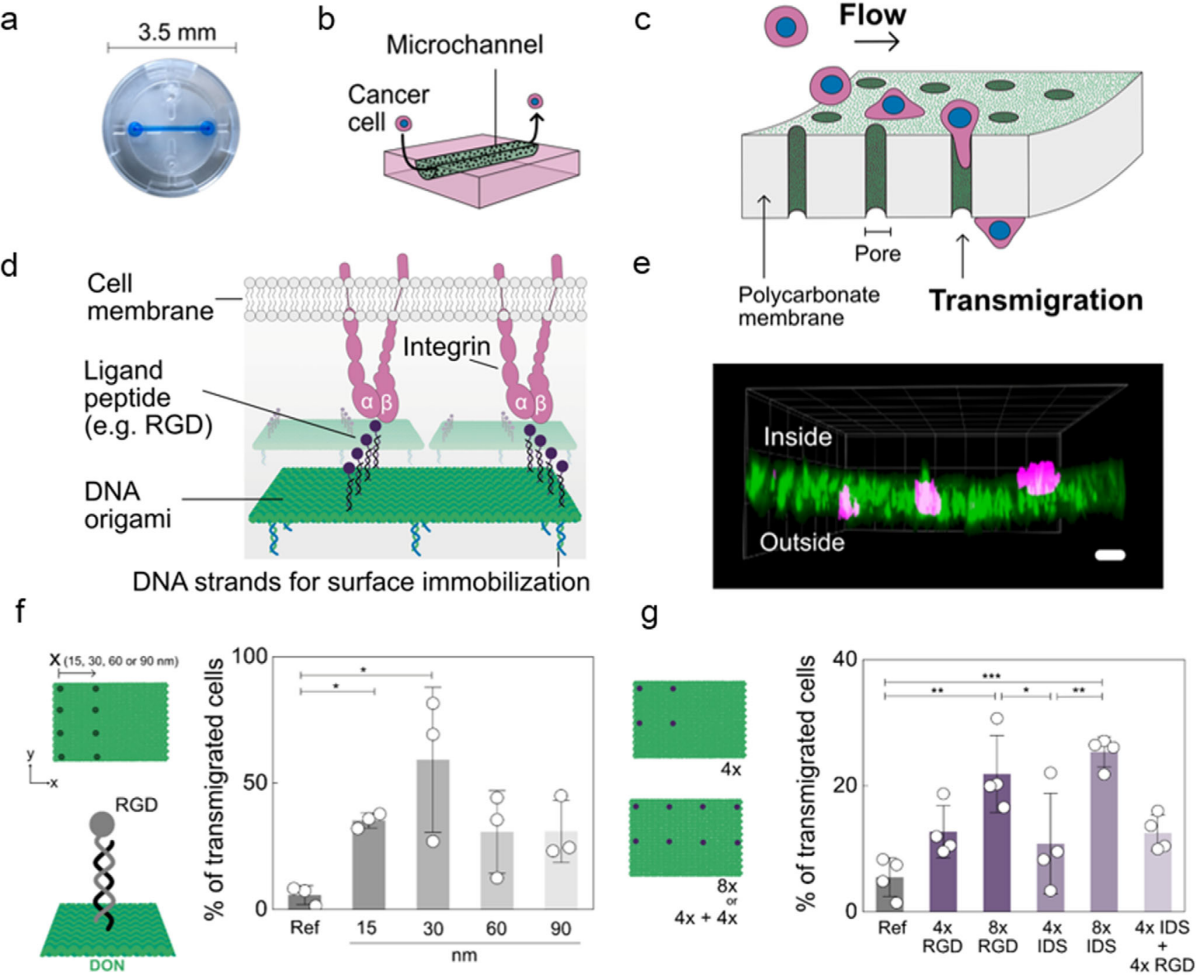
DON‐based study of Integrins.^[^ ^25c^ **^]^** a) Picture b) and schematics of the extravasation device with a porous microchannel (colored blue in the picture) with circulating cancer cells. The chip allows the porous PC membrane to be placed on top of a reservoir into which cells can extravasate. c) Schematic representation of the transmigration of cancer cells through the porous membrane decorated with DONs employed to mimic the endothelial layer. d) Depiction of the DON presenting nanopatterns of RGD‐DNA conjugates. RGD peptides are integrin‐binding ligands and facilitate cell adhesion to the porous flow channel. e) 3D image of MDA‐MB‐231 cells in different positions across the microchannel walls. Actin filaments and nuclei of MDA‐MB‐231 cells are stained with Alexa Fluor 488 Phalloidin and Hoechst, respectively. Scale bar 15 µm. f) Transmigration efficiency obtained with DONs presenting columns of DNA‐RGD conjugates spaced by 15, 30, 60, or 90 nm. g) Transmigration efficiency as a function of the ligand density and combination of RGD and IDS ligands. Reproduced with permission.^[^ ^25c^ ^]^

Integrins recognize mainly extracellular matrix ligands and cell‐surface ligands, with the RGD sequence (arginylglycylaspartic acid) identified as a general integrin‐binding motif for RGD‐recognizing integrins.^[^ ^64^ ^]^ Previous studies by the Spatz group, employing block copolymer micelle nanolithography, suggested a universal optimal spacing of ≈60–70 nm between RGD ligands for maximizing integrin‐mediated cell attachment and spreading.^[^ ^14a,c^ ^]^ However, more recent findings highlight the specificity of spatial distributions depending on integrin subtypes and cell lines.^[^ ^65^ ^]^ For instance, clusters of activated α5β1 integrins exhibited intermolecular distances ranging from ≈5 to 30 nm in HUVEC, CHO, and HeLa cells. Integrin clustering is particularly important for the adhesion of circulating tumor cells (CTCs) to the endothelial walls during extravasation—the process by which CTCs transmigrate across the endothelium to reach the underlying tissue.^[^ ^66^ ^]^ This step is vital for metastatic progression, as it enables tumor cells to exit the bloodstream and establish secondary tumors in distant organs. Building on previous knowledge, García‐Chamé et al. developed an extravasation platform to investigate the effect of integrin clustering on the transmigration capabilities of metastatic breast carcinoma cells (**Figure** **5**a–e). Here, DON decorated with cyclic RGD ligands were used to coat porous microfluidic channels, allowing flowing MDA‐MB‐231 cells to adhere firmly to the channel walls via RGD‐binding integrins and facilitating their extravasation into a secondary chamber devoid of shear stress. Among the tested inter‐ligand distances (15, 30, 60, and 90 nm), 30 nm was found to maximize extravasation efficiency (Figure 5f). Additionally, increasing ligand stoichiometries (8 ligands per DON versus 4 ligands per DON) enhanced extravasation capabilities (Figure 5g). Interestingly, combining different ligands targeting multiple integrin subtypes at 30 nm spacing and equal stoichiometry did not produce a synergistic effect in promoting extravasation within this setup (Figure 5g). Collectively, these findings underscore that optimal integrin clustering is highly dependent on subtype‐specific intermolecular distances, positioning DON technologies as an ideal tool to further investigate these dependencies.

The relatively short cytoplasmic tails of integrins (typically spanning only 20–70 amino acids)^[^ ^67^ ^]^ serve as docking platforms for a surprisingly large and diverse set of intracellular proteins.^[^ ^68^ ^]^ More than 250 proteins have been associated with the formation of integrin adhesion complexes, reflecting a highly intricate network of interactions among them.^[^ ^69^ ^]^ This “integrin cytoplasmic interactome” links integrins to the cytoskeleton and orchestrates downstream signaling pathways that regulate key aspects of cell behavior. However, owing to the complexity of this interactome, the overall picture remains incomplete. For instance, it is not fully understood how is binding specificity achieved, enabling particular adaptor and signaling proteins to access the tail regions in a timely and spatially controlled manner, despite the abundance of potential interactors; or if specific interactome assemblies could be linked to the spatial organization of integrin clusters, which varies with integrin subtype and cell context. Here too, precise ligand presentation on DNA origami enables tailored ligand architectures that function as specific cellular triggers and open up systematic analysis of signaling responses through multi‐omics approaches.

Integrin signaling is deeply interconnected with signaling from growth factor receptors (GFRs) and oncogenic pathways.^[^ ^70^ ^]^ In particular, integrins cooperate closely with EGFR, playing a pivotal role in modulating signaling events at cell adhesion sites.^[^ ^71^ ^]^ Palma´s group has investigated the cooperative effect of integrin and EGFR binding in promoting cancer cell spreading.^[^ ^72^ ^]^ To this end, arrays produced by focused ion beam lithography were used to precisely immobilize single DONs on surfaces to simultaneously display A20FMDV2 peptides, spaced 60 nm apart, alongside EGF to bind αvβ6 integrins and EGFR, respectively. The study examined the attachment and spreading of two human melanoma cell lines, A375Ppuro and A375Pβ6, which differ only in their expression of integrin αvβ6. The results revealed positive cooperation between A20FMDV2 and EGF in promoting the spreading of A375Pβ6 cells. Moreover, it was demonstrated that varying the peptide‐to‐EGF ratio significantly influenced cell spreading outcomes. Nonetheless, further research is needed to elucidate the specificity of the cooperation between particular integrins and GFRs, as well as the detailed molecular mechanisms underlying this crosstalk. As with other signaling pathways, ligand‐decorated DONs represent a highly suitable platform for studying phenomena such as receptor co‐clustering.

### 3.4 Tumor Necrosis Factor Receptor Superfamily (TNFRSF)

Receptors of the tumor necrosis factor receptor superfamily (TNFRSF) are activated by ligands from the TNF superfamily (TNFSF). These receptors play diverse roles in inducing cell death, as well as in regulating inflammation, proliferation, differentiation, and cell migration.^[^ ^73^ ^]^ There are 19 TNFSF ligands known in the family, which are typically trimeric transmembrane proteins but can be cleaved proteolytically to release soluble trimeric ligands. Membrane‐bound ligands recruit three TNFR molecules and initiate the further formation of larger clusters to fully activate the receptors.^[^ ^74^ ^]^ Based on their response to soluble ligands, TNFRs are classified into two categories. Category I receptors are activated effectively by soluble trimers, while category II receptors are not. Rather, they require multimeric presentation of physically bound trimers or their arrangement on a competent surface for activation.^[^ ^73^ ^]^ The TRAIL ligand (TNF‐related apoptosis‐inducing ligand) is initially produced as a ≈40 kDa membrane‐bound protein. Following proteolytic cleavage, it is released as a ≈24 kDa soluble monomer, which assembles into homotrimers that bind to its receptors.^[^ ^75^ ^]^ TRAIL receptors, TRAILR1 (also known as death receptor 4, DR4) and TRAILR2 (also known as death receptor 5, DR5), belong to category II. Clinical trials using agonists to activate these receptors have failed to induce cancer cell death effectively.^[^ ^76^ ^]^

**Figure 6:**
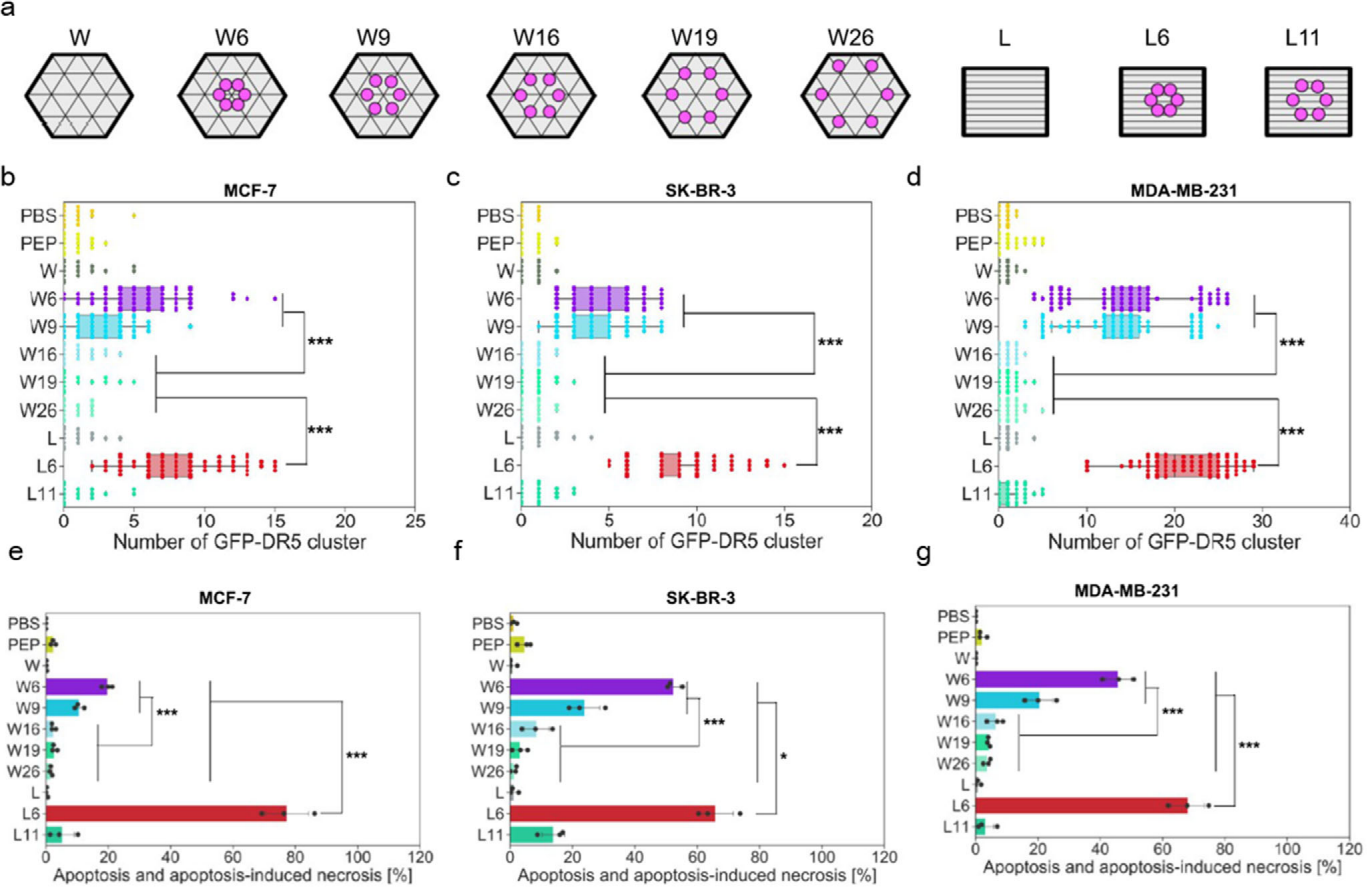
DON‐based study of TNFR.^[^ ^25f^ ^]^ a) DONs containing TRAIL peptidomimetics in hexagonal arrangements with different intermolecular spacings. Naked DONs were also used as controls. b) GFP‐DR5 cluster counting of MCF‐7 cells, c) SK‐BR‐3 cells, and d) MDA‐MB‐231 cells. PBS stands for phosphate‐buffered saline, and PEP stands for peptide. e–g) Percentages of apoptosis and apoptosis‐induced necrosis for e) MCF‐7, f) SK‐BR‐3, and g) MDA‐MB‐231 cells. Adapted with permission.^[^ ^25f^ ^]^

Wang and coworkers tackled the challenge of ineffective activation of category II TRAIL receptors by soluble agonists using nanoscale hexagonal networks of a cyclic peptide that mimics TRAIL's function on DONs (**Figure** **6**a) to induce DR5 clustering by either dimerization of DR5 trimers or trimerization of DR5 dimers, ultimately triggering apoptosis.^[^ ^25f^ ^]^ The bioactive DONs were tested on breast cancer cell lines with varying sensitivities to TRAIL, namely MDA‐MB‐231, MCF‐7, and SK‐BR‐3. By presenting ligands with intermolecular spacings ranging from ≈6 to 26 nm, the authors determined that a ≈6 nm spacing in hexagonal patterns was critical for effective DR5 clustering and cell death (Figure 6b–g). Notably, the findings demonstrated that hexagonal receptor clustering with sub‐10 nm ligand spacing can bypass the previously observed TRAIL resistance of MCF‐7 cells, effectively inducing apoptosis in both TRAIL‐sensitive and resistant breast cancer cells.

Another ligand employed on DONs to target a category II TNFRSF receptor is the Fas ligand (FasL, 45 kDa membrane bound, 26 kDa soluble form), which binds to the Fas receptor (FasR, also known as CD95). Binding of FasL to FasR is also believed to induce the formation of hexagonal supramolecular structures.^[^ ^77^ ^]^ Berger et al. used DONs featuring hexagonal patterns of FasL with 10 nm inter‐ligand spacing. These reagents proved to be the most efficient in initiating apoptosis in HeLa cells, outperforming both homogeneously distributed FasL on surfaces and soluble FasL.^[^ ^78^ ^]^ Deviations from this optimal spacing—whether higher (30 nm) or lower (5 nm)—failed to effectively activate the receptor and induce apoptosis. Interestingly, inter‐ligand spacing had a greater influence on apoptotic efficiency than ligand stoichiometry, as two FasL molecules spaced by the optimal 10 nm elicited stronger apoptotic responses compared to hexamers with suboptimal spacing. These studies highlight that oriented and spatially pre‐clustered TNFLs exhibit maximal efficiency in inducing cell death signaling. Notably, in both cases, the rigidity of ligand presentation, achieved either by enhancing the rigidity of the DON itself^[^ ^25f^ ^]^ or by restricting the flexibility of the linker between the ligand and the DON^[^ ^78^ ^]^ played a crucial role in robust signal initiation.

A recent in vivo study built on these evidences to effectively induce apoptosis of activated immune cells within inflamed synovial tissues, thereby alleviating inflammation and promoting localized immune tolerance in a rheumatoid arthritis mice model.^[^ ^79^ ^]^ The authors employed DONs decorated with hexagonally patterned FasL, featuring 10 nm intermolecular spacing. Notably, the DONs incorporated a pH‐sensitive DNA “lock” mechanism that allowed the origami to close into a hollow cylinder. This structure remained closed under physiological pH (≈7.4) but selectively opened in the slightly acidic environment of inflamed synovial tissue, minimizing off‐target effects such as hepatotoxicity in healthy liver cells.

## 4 Concluding Remarks and Outlook

Receptor clustering is a highly complex process that has become a central focus in the study of fundamental biological mechanisms using DNA nanotechnology over the past decade. This review highlights, through representative examples, the exceptional suitability of DNA origami platforms for investigating the initiation and impact of receptor clustering on cellular function across different contexts, cell types, and effector functions. The examples clearly demonstrate that DNA nanostructures functionalized with receptor ligands can efficiently and precisely generate nanoscale ligand patterns and present them to a wide range of cell types. The findings obtained so far already provide valuable insights into receptor behavior, which, as shown here, varies significantly depending on the receptor type.

However, challenges remain that must be addressed to fully unlock the potential of this promising approach and achieve a transformative impact on both fundamental and applied biomedical research. Key areas for improvement include (i) automation by developing high‐throughput systems for DNA origami synthesis, biofunctionalization, delivery and cell response observation; (ii) implementation of in‐depth signaling pathway studies to explore the downstream effects of receptor clustering across diverse biological systems; (iii) combination studies to investigate how multiple receptor types interact and influence clustering and signaling; (iv) investigating how the spatial arrangement, combination, and presentation of ligands within the 2D and 3D architecture of DONs influence receptor activation; and last but not least (v) standardization and rigorous research data management including stringent design and implementation of controls to ensure robust and reproducible experimental designs to validate findings. In fact, the establishment of standardized operating procedures (SOPs) for experiments and analyses seems the most critical and currently missing task in the field. E.g., there is currently no standardized terminology or methodology for calculating ligand occupancy, a key parameter that defines the functionalization of DONs with bioactive groups, which is crucial for the resulting biological response. Current practices often vary, and to accurately represent the statistical distribution of ligand occupancy, detailed histograms should be used instead of a single numerical value. Implementing a standardized approach would not only enhance clarity, but also significantly improve comparability between studies. With such standardization in place, it would be particularly valuable to systematically compare the effects of different DON shapes, display and delivery systems on signal initiation and transduction, allowing for the identification of optimal formats for specific investigations.

By addressing the challenges, DNA‐based biomimetic nanoarrays have a huge potential to drive breakthroughs in biomedicine. These platforms could for instance influence the design of therapeutic antibodies that rely on FcγR or FcεRI engagement, potentially leading to more effective therapies for cancer, immune disorders, and neurodegenerative diseases, or novel vaccines. E.g., the promising results related to the IR and TNFR underscore their therapeutic potential. Multivalent insulin presentation on DONs demonstrated superior efficacy compared to unmodified insulin at the same concentration, paving the way for more potent insulin formulations. Additionally, while soluble TNFR agonists have failed to induce cancer or immune cell death, their multivalent presentation on DONs successfully triggered apoptotic responses. These findings open new avenues to enhance diabetes treatment efficacy, overcome resistance to current TNFRSF‐targeting drugs, and develop safer—less hepatotoxic—therapeutic strategies for autoimmune diseases such as rheumatoid arthritis.

In conclusion, DNA origami technology offers a groundbreaking platform for unraveling the complexities of receptor clustering, with far‐reaching implications for cell biology and therapeutic innovation. By overcoming the existing challenges, this field has the potential to revolutionize biomedical research, paving the way for deep insights into cellular signaling and precision‐targeted therapies.

## Conflict of Interest

The authors declare no conflict of interest.
